# Tannic Acid Promotes TRAIL-Induced Extrinsic Apoptosis by Regulating Mitochondrial ROS in Human Embryonic Carcinoma Cells

**DOI:** 10.3390/cells9020282

**Published:** 2020-01-23

**Authors:** Nipin Sp, Dong Young Kang, Eun Seong Jo, Alexis Rugamba, Wan Seop Kim, Yeong-Min Park, Dae-Yong Hwang, Ji-Seung Yoo, Qing Liu, Kyoung-Jin Jang, Young Mok Yang

**Affiliations:** 1 Department of Pathology, School of Medicine, Institute of Biomedical Science and Technology, Konkuk University, Seoul 05029, Korea; nipinsp@gmail.com (N.S.); kdy6459@naver.com (D.Y.K.); eses0706@naver.com (E.S.J.); rugambalex@gmail.com (A.R.); wskim@kuh.ac.kr (W.S.K.); 2 Department of Immunology, School of Medicine, Konkuk University, Chungju 27478, Korea; immun3023@kku.ac.kr; 3 Department of Surgery, School of Medicine, Konkuk University, Seoul 05029, Korea; hwangcrc@kuh.ac.kr; 4 Department of Immunology, Hokkaido University Graduate School of Medicine, Sapporo 060-0808, Japan; jiseungy@pop.med.hokudai.ac.jp; 5 Jilin Green food Engineering Research Institute, Changchun 130000, Jilin, China; liuqing0523@hotmail.com

**Keywords:** Tannic acid, Wnt/β-catenin, mitochondrial ROS, TRAIL, extrinsic apoptosis pathway

## Abstract

Human embryonic carcinoma (EC; NCCIT) cells have self-renewal ability and pluripotency. Cancer stem cell markers are highly expressed in NCCIT cells, imparting them with the pluripotent nature to differentiate into other cancer types, including breast cancer. As one of the main cancer stem cell pathways, Wnt/β-catenin is also overexpressed in NCCIT cells. Thus, inhibition of these pathways defines the ability of a drug to target cancer stem cells. Tannic acid (TA) is a natural polyphenol present in foods, fruits, and vegetables that has anti-cancer activity. Through Western blotting and PCR, we demonstrate that TA inhibits cancer stem cell markers and the Wnt/β-catenin signaling pathway in NCCIT cells and through a fluorescence-activated cell sorting analysis we demonstrated that TA induces sub-G1 cell cycle arrest and apoptosis. The mechanism underlying this is the induction of mitochondrial reactive oxygen species (ROS) (mROS), which then induce the tumor necrosis factor-related apoptosis-inducing ligand (TRAIL)-mediated extrinsic apoptosis pathway instead of intrinsic mitochondrial apoptosis pathway. Moreover, ribonucleic acid sequencing data with TA in NCCIT cells show an elevation in TRAIL-induced extrinsic apoptosis, which we confirm by Western blotting and real-time PCR. The induction of human TRAIL also proves that TA can induce extrinsic apoptosis in NCCIT cells by regulating mROS.

## 1. Introduction

Human embryonic carcinoma (EC) cells exhibit similar gene expression profiles to those of embryonic stem (ES) cells, as both have an unlimited self-renewal ability and the capability to differentiate into all derivatives of the three embryonic germ layers—the ectoderm, endoderm, and mesoderm [1,2,3]. As such, these cells can develop into several cell types in the adult human body [4]. Sex-determining region Y (SRY)-box 2 (SOX2), octamer-binding transcription factor 4 (OCT4), and homeobox protein NANOG are considered the stem cell markers expressed in ES and EC cells that help maintain the pluripotent ES cell phenotype [5,6]. Hyper-expression of these stem cell markers leads to the aberrant self-renewal of ES cells, which can promote oncogenesis [7,8]. Among them, overexpression of SOX2 results in abnormal stem cell self-renewal in breast cancer cells [9,10], which aids in tumor progression and contributes to poor clinical outcomes in breast cancer [11].

Regulation of EC cells helps cancer chemoprevention in terms of treatment with phytochemicals during cancer prevention or cancer recurrence stages to induce apoptotic cell death in cancer stem cells [12,13]. The use of dietary phytochemicals originating from edible foods for cancer chemoprevention is a good method, as these natural substances lack side effects after treatment [14,15]. Tannic acid (TA) is a polyphenol originating from plants that is usually found in tea, red wine, nuts, beans, vegetables, and wood bark [16]. It has anti-cancer activity against a wide spectrum of cancers, including chemically-induced cancers [17,18,19,20], and can also act against cancer stem cells via inhibition of the NF-κB-mediated phenotype transition of breast cancer cells [21].

The Wnt/β-catenin pathway is one of the primary molecular pathways that help cancer stem cells with their self-renewal and pluripotency behavior. The Wnt family of secreted glycolipoproteins and its transcription co-activator factor, β-catenin, actively take part in embryonic development and homeostasis in canonical Wnt signaling [22]. In Wnt signaling, the phosphorylation of β-catenin is mainly regulated by casein kinase 1α and glycogen synthase kinase 3β (GSK-3β) [23], which then leads to proteasomal degradation or ubiquitination. It can bind to DNA sequences specific to the transcription factor (TCF)-binding element in TCF/lymphoid enhancer-binding factor (LEF) present in the nucleus to promote the transcription process [24]. Elevated signaling of Wnt/β-catenin occurs more often in triple-negative breast cancer (TNBC) compared with other breast cancers [25,26]. Thus, targeting the Wnt/β-catenin signaling in TNBC for phytochemical treatment is a good choice, as such a treatment also targets breast cancer stem cells [27].

The induction of apoptosis in breast cancer prevents cancer progression, but there is a chance of cancer recurrence if the breast cancer stem cells are still present in the system. Targeting cancer stem cells instead of cancer cells is a better method of fighting against cancer. The tumor necrosis factor-related apoptosis-inducing ligand (TRAIL) can induce death signaling by binding to its death receptors (DRs), TRAIL-receptor 1 (TRAIL-R1)/death receptor 4 (DR4), and TRAIL-R2/DR5 [28,29]. These DRs also have a cytoplasmic death domain that contains their adaptor protein—tumor necrosis factor (TNF) receptor-associated death domain (TRADD) protein. The interaction of TRADD with DR4 or DR5 triggers a variety of cell signals, including the activation of caspases [30]. In the extrinsic pathway of apoptosis, TRAIL signaling activates caspase-8 or caspase-10, which then initiates apoptosis by cleaving and activating executioner caspase-3, caspase-6, and caspase-7 [31]. This TRAIL could be used as a therapeutic target for chemotherapy in TNBC, as they are sensitive to TRAIL, leading to TRAIL-induced apoptosis [32,33].

Reactive oxygen species (ROS) are free radicals (i.e., ions or molecules that occur in an elevated state in most cancers) [34]. The mitochondria are considered a major source of ROS, wherein ~2% of O_2_ is diverted to mitochondrial ROS (mROS) [35], which actively takes part in cell signaling as well as cell death signaling [36]. This ROS can induce the intrinsic pathway of apoptosis by enhancing DNA damage [37]. Some studies have shown that ROS is actively involved in the apoptosis in TRAIL-treated cancer cells [38,39]. The induction of apoptotic signals includes the formation of mROS. The downregulation of the mitochondrial membrane potential leads to the release of pro-apoptotic proteins, and membrane depolarization and TRAIL induction may also occur as a result of mROS [40,41].

In this study, we hypothesize that TA can induce TRAIL-mediated apoptosis in NCCIT ES cell carcinoma. We also aim for the induction of mROS by TA to aid the TRAIL-induced extrinsic pathway of apoptosis in NCCIT cells.

## 2. Materials and Methods

### 2.1. Antibodies and Cell Culture Reagents

Roswell Park Memorial Institute-1640 (RPMI-1640) medium, penicillin-streptomycin solution, and trypsin-EDTA (0.05%) were purchased from Gibco (Thermo Fisher Scientific, Inc., Waltham, MA, USA). Tannic acid (TA; Cas. No: 1401-55-4), fetal bovine serum (FBS; 12003C), Zb (Z3902), and primary antibodies specific for SOX2 (MAB4423), OCT4 (MABD76), and NANOG (MABD24) were purchased from Sigma-Aldrich (Merck KGaA, St. Louis, MO, USA). Antibodies specific for β-actin (sc-47778), TRAIL (sc-8440), TRADD (sc-46653), Wnt5A (sc-365370), Casp8 (sc-81656), Bcl-2 (sc-7382), p21 (sc-756), cyclin E (sc-481), and CDK4 (sc-260) and secondary antibodies (anti-mouse (sc-516102) and anti-rabbit (sc-2357)) were obtained from Santa Cruz Biotechnology, Inc. (Dallas, TX, USA). The Wnt8A (H00007478-B01P) antibody was obtained from Abnova (Taipei City, Taiwan); pEGFR (#2234), β-Catenin (#9582), GSK-3β (#9315), Bax (#2772), Casp9 (#9502), C-Casp9 (#9505), Casp3 (#9662), C-Casp3 (#9661), cytochrome C (#11940), and p27 Kip1 (#3686) antibodies and a TCF/LEF Family Antibody Sampler Kit (#9383) were purchased from Cell Signaling Technology, Inc. (Beverly, MA, USA). Finally, DR4 (ab8415), DR5 (ab8416), and cyclin D1 (ab6152) antibodies were purchased from Abcam (Cambridge, MA, USA).

### 2.2. Cell Culture and Treatment

The NCCIT (CRL-2073, ATCC Manassas, VA, USA) human ES cells were cultured in RPMI-1640 supplemented with 10% FBS and 1% penicillin and streptomycin at 37 °C in 5% CO_2_. For each experiment, at 80% confluence, the cells were gently washed twice with phosphate-buffered saline (PBS). Unless otherwise specified, the cells were treated with various concentrations of TA for different time periods according to the experiment pattern at 37 °C.

### 2.3. Cell Proliferation Inhibition

Cell proliferation inhibition was carried out using a crystal violet assay. The NCCIT cells were seeded in six-well plates and incubated overnight under ambient conditions. After 24 h of incubation, the cells were treated with increasing concentrations of TA (5–100 µM) for 24 or 48 h. The cell proliferation was then analyzed using crystal violet at 560 nm.

### 2.4. Western Blotting

Whole cell lysates were prepared from untreated or TA-treated NCCIT cells by incubating them on ice with radio immunoprecipitation lysis buffer (20-188; EMD Millipore) containing phosphatase and protease inhibitors to isolate protein. The protein concentrations were measured via the Bradford method (Thermo Fisher Scientific, Inc., Waltham, MA, USA). Equal amounts of protein (100 μg/well) were resolved with 10% SDS-PAGE. Then, the separated proteins were transferred onto nitrocellulose membranes. The blots were blocked for 1 h with 5% skim milk (BD Biosciences, San Jose, CA, USA; 90002-594) in TBS-T buffer (20 mM Tris-HCl (Sigma-Aldrich; Merck KGaA, St. Louis, MO, USA; 10708976001), pH 7.6, 137 mM NaCl (Formedium, Norfolk, UK; NAC03), 0.1X Tween 20 (Scientific Sales, Inc. Oak Ridge, TN, USA; 0777)). The membranes were then incubated overnight at 4 °C in a shaker with primary antibodies diluted in 5% bovine serum albumin (EMD Millipore). The membranes were then washed with TBS-T and incubated for 1 h at room temperature with Horseradish peroxidase (HRP)-conjugated secondary antibodies. Detection was performed with a Femto Clean Enhanced Chemiluminescence Solution Kit (GenDEPOT; 77449; Katy TX) and a LAS-4000 imaging device (Fujifilm, Tokyo, Japan).

### 2.5. Reverse Transcription-Polymerase Chain Reaction (RT-PCR)

Total RNA was extracted with the RNeasy Mini Kit (Qiagen GmbH, Hilden, Germany) according to the manufacturer’s protocol. The RNA was quantified spectrophotometrically at 260 nm, and cDNA was synthesized from the total RNA at 42 °C for 1 h and at 95 °C for 5 min with a first-strand cDNA synthesis kit (K-2041; Bioneer Corporation, Daejeon, Korea) and oligo d(T) primers. The RT-PCR Premix Kit (K-2016; Bioneer Corporation) was used to amplify SOX2, OCT4, NANOG, and GAPDH with primers synthesized by the Bioneer Corporation. The PCR conditions were as follows: 95 °C for 5 min, followed by 32 cycles at 95 °C for 60 s, 58 °C for 60 s, 72 °C for 60 s, and then, 72 °C for 10 min. The primers used for the amplification are listed in Table S1. The PCR products were resolved by electrophoresis on 1.5% agarose gel and visualized with ethidium bromide (E7637; Sigma-Aldrich; Merck KGaA) staining.

### 2.6. RNA Sequencing Analysis

Total RNA was extracted using Trizol reagent (Invitrogen, Carlsbad, CA, USA). Isolated RNA quality was analyzed by Agilent 2100 bioanalyzer using RNA 6000 Nano Chip (Agilent Technologies, Amstelveen, The Netherlands). RNA quantification was done by ND-2000 Spectrophotometer (Thermo Inc., Wilmington, DE, USA). For control and TA treated RNAs, the construction of a library was done by QuantSeq 3′ mRNA-Seq Library Prep Kit (Lexogen, Inc., Vienna, Austria) according to the manufacturer’s protocol. Each 500 ng of TA treated and non-treated total RNA was prepared and an oligo-dT primer containing a 5′ Illumina-compatible sequence was hybridized to the RNA and reverse transcription was carried out. After RNA template degradation, initiation of second strand synthesis was conducted using a random primer that contained a 5′ end Illumina-compatible linker sequence. Magnetic beads were used to purify the double-stranded library to remove all reaction components. For cluster generation, the library was amplified to add the complete adapter sequences. The final library was purified from PCR components. A single-end 75 sequencing was carried out as a high-throughput sequencing by using NextSeq 500 (Illumina, Inc., San Diego, CA, USA). QuantSeq 3′ mRNA-Seq reads were aligned by Bowtie2. Differentially expressed genes were determined by the counts from alignments using coverage in Bedtools. The Read Count data were processed based on quantile normalization method by EdgeR within R (R development Core Team, 2016) using a bioconductor. Gene classification was made based on searches performed in DAVID and Medline databases.

### 2.7. Quantitative Polymerase Chain Reaction (Real-Time qPCR)

A real-time PCR was performed in a thermal cycler (C1000 Thermal Cycler, Bio-Rad, Hercules, CA, USA), as follows: 2 μL diluted cDNA was added to 10 μL TB Green Advantage Premix (Takara Bio, Japan) and 1 μL each of 100 pM of diluted forward and reverse primers. The conditions used for the real-time qPCR were as follows: initial denaturation at 95 °C for 5 min, followed by 40 cycles of denaturation at 95 °C for 40 s, annealing at 58 °C for 40 s, extension at 72 °C for 40 s, and a final extension at 72 °C for 5 min. The primers used for the amplification are listed in Table S2. All measurements were performed in triplicate. The relative expression of the target genes was normalized to GAPDH. The calculations were carried out using the Cp values.

### 2.8. Cell Cycle Analysis

The DNA content of TA treated and non-treated cells was determined by a BD Cycletest Plus DNA Reagent Kit (BD Biosciences, San Jose, CA, USA) according to the manufacturer’s protocol. Approximately 5 × 10^5^ cells, with or without TA for 24 h or 48 h, were washed with PBS and permeabilized with trypsin. The neutralization of RNA interaction with propidium iodide (PI) was done by treating the cells with RNase buffer and trypsin inhibitor. The samples were then stained with PI and incubated for 30 min in the dark at room temperature and analyzed by a FACSCalibur flow cytometer (BD Biosciences, San Jose, CA, USA).

### 2.9. Apoptosis Analysis

Fluorescein-conjugated annexin V (annexin V-FITC) was used to measure the apoptosis in NCCIT cells. The TA- or Zb-treated cells were washed with PBS and re-suspended in a binding buffer at a concentration of 1 × 10^6^ cells. Then the cells were stained with annexin V-FITC and PI for 10 min in the dark at room temperature. The percentage of apoptotic cells was measured by flow cytometry via FACSCalibur and the analysis was performed using FlowJo software.

### 2.10. Fluorescence-Activated Cell Sorting (FACS) Analysis for Mitochondrial Membrane Potential and ROS

After the cultured cells were washed with prewarmed no-glucose RPMI-1640 medium (11879020; Gibco) supplemented with 10% FBS (staining buffer), 1 × 10^6^ cells were resuspended in 1 mL of staining buffer containing MitoTracker DeepRed (40 nM; M22426; Invitrogen) for mitochondrial membrane potential and MitoSOX (5 μM; M36008; Invitrogen) for mROS. Then, the cells were incubated in a CO_2_ incubator at 37 °C for 30 min. The stained cells were washed with 1 mL of prewarmed staining buffer and used for FACS analysis. The analysis was performed using FlowJo software.

### 2.11. Caspase-Glo 3/7 Assay

This method was performed using the Caspase-Glo^®^ 3/7 Assay System from Promega (G8090; Fitchburg, WI, USA). The NCCIT cells were seeded (20,000 cells/well) in a white-walled 96-well plate and treated with TA after reaching 80% confluence. After incubation with TA, Caspase-Glo^®^ 3/7 Reagent was added to each well and incubated in a plate shaker at 500 rpm for 3 h. After incubation, readings were taken using a plate-reading luminometer, and calculations were done according to assay protocol.

### 2.12. ATP Determination Assay

This method was performed using an ATP Determination Kit from Molecular Probes (A22066; Eugene, OR, USA). Briefly, NCCIT cells were treated with TA, and an equal number of cells was collected for the ATP determination assay. The standard reaction solution for the samples was made using reaction buffer, Dithiothreitol (DTT), D-luciferin, and firefly luciferase provided in the kit; the cells were added along with the standard reaction solution. After incubation, readings were taken using a plate-reading luminometer, and calculations were done according to assay protocol.

### 2.13. Human TRAIL Enzyme-Linked Immunosorbent Assay (ELISA)

This method was performed via the ELISA for quantitative detection using a Human TRAIL ELISA Kit from Invitrogen (BMS2004; Carlsbad, CA, USA). The NCCIT cells were treated with Zb and TA for 48 h, and spent media were used for the assay. The samples were added to anti-human TRAIL-coated microwells along with sample diluent and a biotin-conjugate solution. After incubation, streptavidin-HRP was added and further incubated; 3,3′,5,5′-Tetramethylbenzidine (TMB) solution was added after washing. Finally, a stop solution was added once the high-concentrated standard turned a dark blue color. The absorbance was read at 450 nm, and calculations were performed according to assay protocol.

### 2.14. Statistical Analyses

All experiments were performed at least three times. The results were expressed as the mean ± standard error of the mean. Statistical analyses were conducted via the one-way analysis of variance (ANOVA) or the Student’s *t*-test. The one-way ANOVA was performed with Tukey’s post hoc test. The analyses were performed with the SAS 9.3 software program (SAS Institute, Inc., Cary, NC, USA). A *p*-value < 0.05 was taken to indicate a statistically significant difference.

## 3. Results

### 3.1. TA Inhibits Cell Proliferation of NCCIT Cells as Well as Cancer Stem Cell Markers

**Figure 1:**
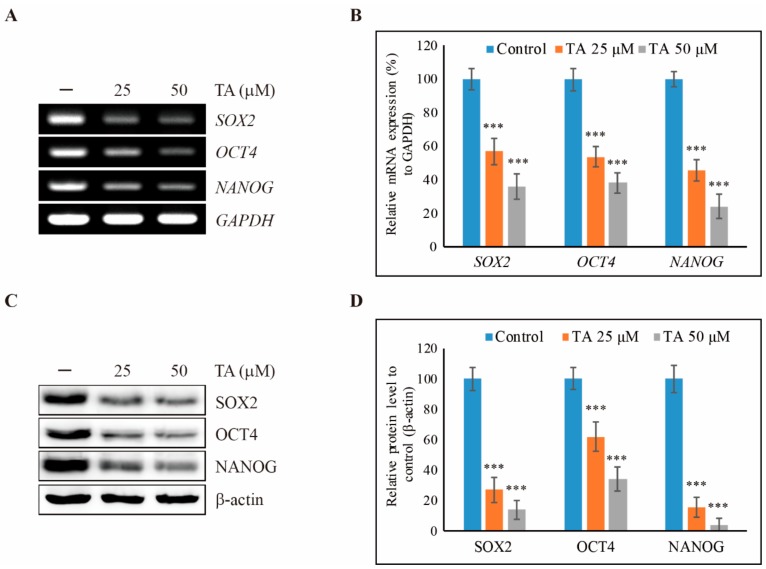
Tannic acid (TA) inhibits cancer stem cell markers in NCCIT cells. (**A**) The expression levels of *SOX2*, *OCT4*, and *NANOG* mRNA in the NCCIT cells were detected after TA treatment in concentrations indicated for 48 h. (**B**) The representative expression levels of mRNA were determined by densitometry and normalized to GAPDH mRNA. Controls are set to 100. Data are representative of three independent experiments. *** *p* < 0.001 (*t*-test). (**C**) Western blotting analysis of NCCIT cells with 25 µM and 50 µM of TA for 48 h showing the inhibition of cancer stem cell markers SOX2, OCT4, and NANOG expressions. (**D**) The representative expressions of proteins were determined via densitometry and normalized to β-actin. Controls are set to 100. Data are representative of three independent experiments. *** *p* < 0.001 (*t*-test).

To determine the cell proliferation inhibition of NCCIT cells by TA, we used a crystal violet assay and compared the effects of TA with non-treated control cells (Figure S1A). The obtained results showed a concentration-dependent inhibition of cell proliferation of NCCIT after 24 h and 48 h of treatment with TA. From this, 50 µM TA was used as the IC_50_ dosage for a time period of 48 h, which we also used for further studies. To determine whether TA plays a role in the inhibition of stem cell markers, we checked the expression of stem cell markers *SOX2, OCT4*, and *NANOG* in the mRNA level and obtained a significant concentration-dependent inhibition of these stem cell markers by TA in the NCCIT cells (Figure 1A,B). Then, we confirmed the stem cell marker inhibition of TA by real-time PCR (Figure S1B). We checked these stem cell marker expression levels in the protein level (Figure 1C) and found that TA inhibited stem cell markers SOX2, OCT4, and NANOG significantly (Figure 1D). 

### 3.2. TA Downregulates the Wnt/β-Catenin Pathway in NCCIT Cells

**Figure 2:**
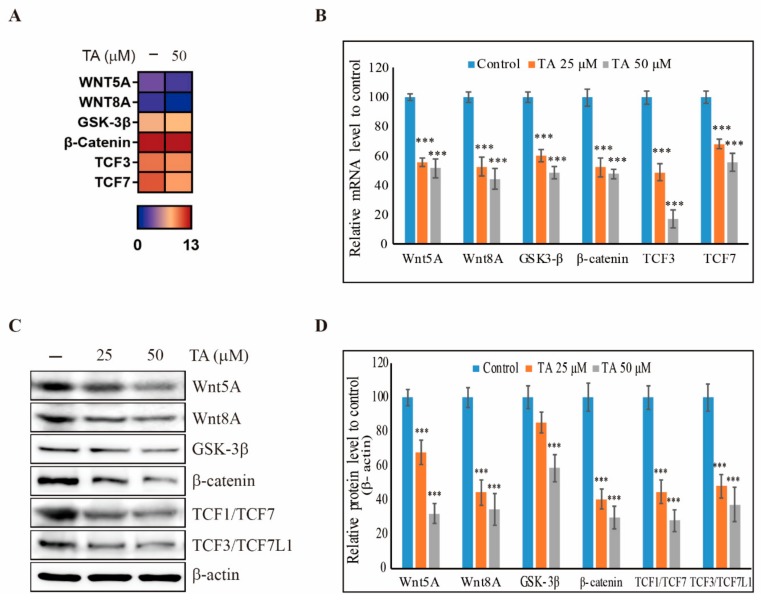
TA inhibits Wnt/β-catenin signaling in NCCIT cells. (**A**) Heat map showing the fold changes relative to the mean expression of Wnt/β-catenin pathway in non-treated and TA-treated NCCIT cells for 48 h. (**B**) Real-time PCR data of mRNA after treatment with TA for 48 h showing the relative expression levels of the Wnt/β-catenin pathway and normalized to GAPDH mRNA. Controls are set to 100. Data are representative of three independent experiments. *** *p* < 0.001 (*t*-test). (**C**) Western blotting analysis of NCCIT cells with 25 µM and 50 µM of TA for 48 h showing the inhibition of Wnt5A, Wnt8A, glycogen synthase kinase 3β (GSK-3β), β-catenin, transcription factor 1 (TCF1)/TCF7, and TCF3/TCF7L1 expressions. (**D**) The representative expressions of proteins were determined via densitometry and normalized to β-actin. Controls are set to 100. Data are representative of three independent experiments. *** *p* < 0.001 (*t*-test).

The TA treatment in NCCIT cells showed an inhibition of stem cell markers, so we investigated the ability of TA to inhibit the Wnt/β-catenin stem cell pathway. Firstly, we analyzed the samples with RNA sequencing (Figure S2A,B) and obtained results suggested an inhibition of Wnt/β-catenin pathway with TA treatment with respect to the control (Figure 2A). We confirmed the inhibition of Wnt/β-catenin pathway by TA in NCCIT cells at the mRNA level by real-time PCR (Figure 2B). Then we confirmed the inhibition of Wnt signaling via the inhibition of the GSK-3β, β-catenin, and TCF signaling cascade in the protein level (Figure 2C). The relative expressions of proteins indicated that TA downregulated the Wnt/β-catenin pathway in a concentration-dependent manner (Figure 2D). 

### 3.3. TA Induces Sub-G1 Cell Cycle Arrest in NCCIT Cells

**Figure 3:**
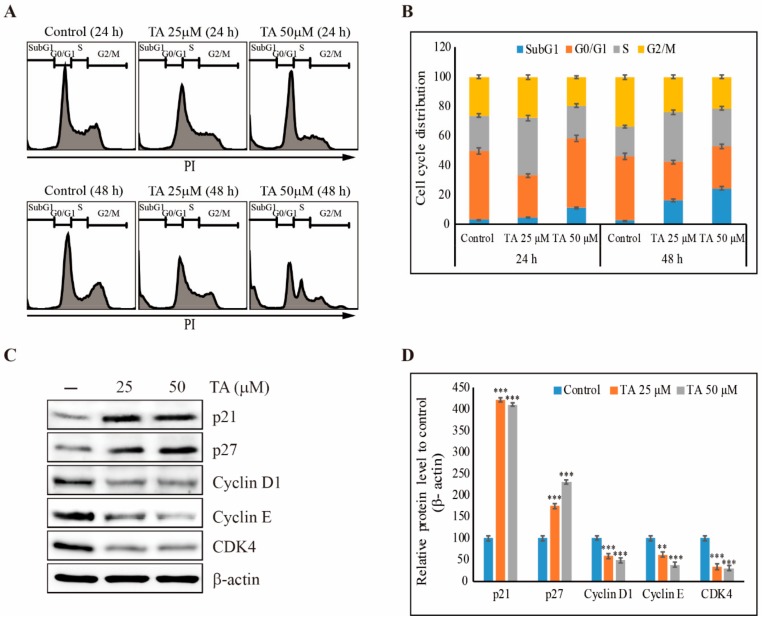
TA induces cell cycle arrest in NCCIT cells. (**A**) Flow cytometry analysis showing sub-G1 arrest after treatment with 25 µM and 50 µM TA for 24 h and 48 h in NCCIT cells. (**B**) Graphical representation of the cell distribution in the sub-G1 phase. Experiments were repeated three times, and mean values are presented in the final graph. (**C**) Western blotting analysis of cell cycle markers after treatment with TA for 48 h. (**D**) The representative expressions of the p21, p27, cyclin D1, cyclin E, and CDK4 proteins were determined by densitometry and normalized to β-actin. Controls are set to 100. ** *p* < 0.01 and *** *p* < 0.001 (*t*-test).

As we found that TA could inhibit the cell proliferation of NCCIT cells, we performed a cell cycle analysis in NCCIT cells with TA treatment. The obtained results showed an increase in the cell count in the sub-G1 phase by TA compared with non-treated control cells (Figure 3A). The TA also helped accumulate cells in the S phase, but not in a concentration-dependent manner (Figure 3B). To confirm the cell cycle arrest, we checked the checkpoint proteins after TA treatment in the protein level (Figure 3C). The results showed an increase in the expression levels of tumor suppressor proteins p21 and p27 and a significant inhibition of cell cycle markers cyclin D1, cyclin E, and CDK4 (Figure 3D).

### 3.4. TA Induces Apoptosis in NCCIT Cells

**Figure 4:**
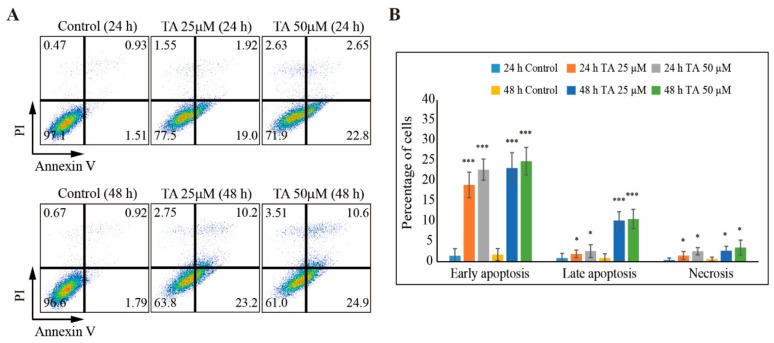
TA induces apoptosis in NCCIT cells. (**A**) Fluorescein-conjugated annexin V (annexin V-FITC) vs. propidium iodide (PI) staining analysis showing apoptosis induction after treatment with 25 µM and 50 µM TA for 24 h and 48 h in NCCIT cells. (**B**) Graphical representation of the percentage of apoptotic cells upon control, 25 µM, and 50 µM TA treatment for 24 h and 48 h obtained from fluorescence-activated cell sorting (FACS) data. * *p* < 0.05 and *** *p* < 0.001 (*t*-test).

The cell cycle arrest in NCCIT cells with TA treatment also indicated the possibility of TA inducing apoptosis. We analyzed NCCIT cells with or without treatment with TA for 24 h and 48 h using fluorescein-conjugated annexin V (annexin V-FITC) and propidium iodide (PI) staining in fluorescence-activated cell sorting (FACS) (Figure 4A). The obtained results demonstrated the induction of early apoptosis by TA at 24 h and some late apoptosis at 48 h (Figure 4B).

### 3.5. TA Induces mROS and Inhibits the Production of ATP in NCCIT Cells

**Figure 5:**
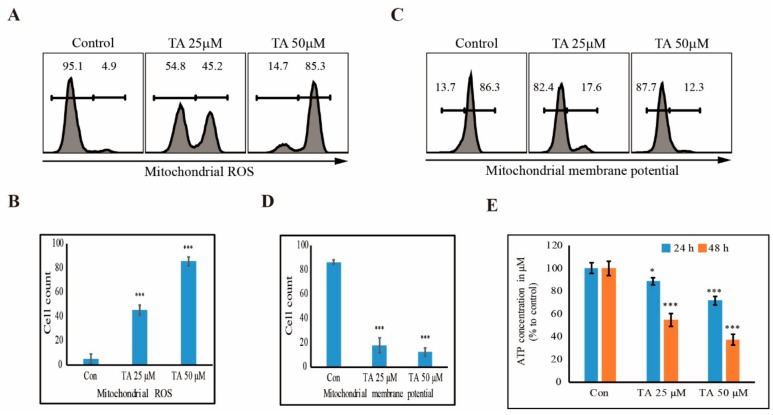
TA induces mROS and inhibits ATP production in NCCIT cells. (**A**) Flow cytometry analysis showing mROS after treatment with 25 µM and 50 µM TA for 48 h in NCCIT cells. (**B**) Graphical representation of mROS production with TA treatment. *** *p* < 0.001 (*t*-test). (**C**) Flow cytometry analysis showing mitochondrial membrane potential after treatment with 25 µM and 50 µM TA for 48 h in NCCIT cells. (**D**) Graphical representation of mitochondrial membrane potential with TA treatment. *** *p* < 0.001 (*t*-test). (**E**) ATP determination assay showing ATP production in NCCIT cells after treatment with 25 µM and 50 µM TA for 24 h and 48 h. Controls are set to 100. * *p* < 0.05 and *** *p* < 0.001 (*t*-test).

After determining that TA induced cell cycle arrest and apoptosis in NCCIT cells, we investigated whether treatment with TA induces ROS formation. We checked the level of mROS and observed that TA treatment increased the production of mROS in a concentration-dependent manner (Figure 5A). The significant induction of mROS suggested the induction of apoptosis by TA (Figure 5B). To confirm the activity of mitochondria in apoptosis, we checked the mitochondrial membrane potential after TA treatment in NCCIT cells (Figure 5C). The results showed a significant decrease in the mitochondrial membrane potential (Figure 5D), indicating the loss of the membrane integrity of the mitochondria, which further indicated apoptosis. Then, we observed the ATP production in NCCIT cells after treatment with TA; the results showed a concentration- and time-dependent inhibition in ATP formation (Figure 5E), suggesting that TA did not induce the mitochondrial apoptosis.

### 3.6. Induction of Extrinsic Pathway of Apoptosis by TA in NCCIT Cells

**Figure 6:**
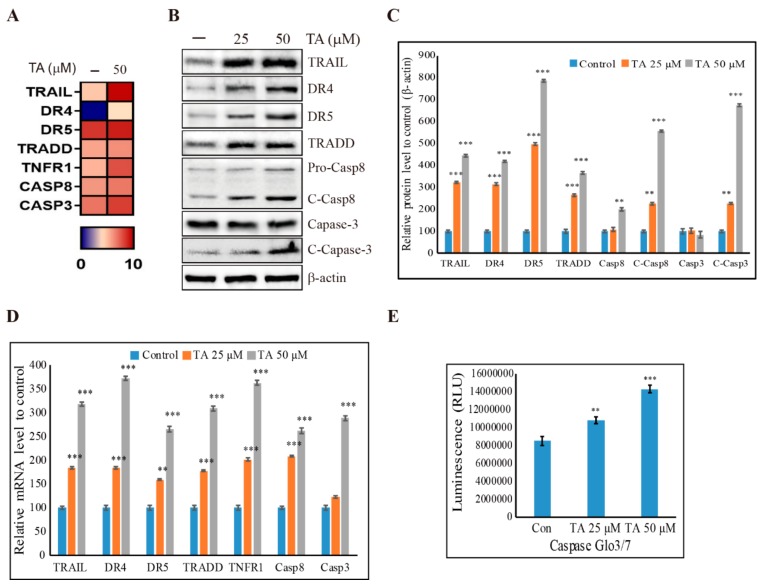
TA induces extrinsic apoptosis in NCCIT cells. (**A**) Heat map showing the fold changes relative to the mean expression of extrinsic apoptosis pathway in non-treated and TA-treated NCCIT cells. (**B**) Western blotting analysis of NCCIT cells with 25 µM and 50 µM of TA for 48 h showing the inhibition of extrinsic apoptotic protein expressions. (**C**) The representative expressions of proteins were determined via densitometry and normalized to β-actin. Controls are set to 100. Data are representative of three independent experiments. ** *p* < 0.01 and *** *p* < 0.001 (*t*-test). (**D**) Real-time PCR data of mRNA after treatment with TA showing the relative expression levels of the extrinsic apoptosis pathway and normalized to GAPDH mRNA. Controls are set to 100. Data are representative of three independent experiments. ** *p* < 0.01 and *** *p* < 0.001 (*t*-test). (**E**) Caspase-Glo 3/7 assay showing the enhancement of caspase-3 and caspase-7 activity with TA treatment for 24 h. ** *p* < 0.01 and *** *p* < 0.001 (*t*-test).

We found that TA induced cell cycle arrest, mROS production, and apoptosis, so we investigated apoptosis pathway induction by TA. First, we analyzed the key factor molecules, BCL2 Associated X (BAX) and B-cell lymphoma 2 (BCL-2) by RNA sequencing (Figure S3A), which showed a decreased expression of BAX and non-significant expressions for BCL-2. This indicated that it is not the intrinsic pathway of apoptosis; thus, we checked the expression patterns of caspase-9 and cytochrome C, which also showed a significant decrease in the expressions (Figure S3B,C). Then, we confirmed these expressions in the mRNA level to confirm that TA did not induce the intrinsic apoptosis pathway (Figure S3D). The BAX/BCL-2 ratio of protein and mRNA also suggested that TA did not induce intrinsic mitochondrial apoptosis (Figure S3E). Following this, we investigated the RNA sequencing for non-treated control cells and cells treated with 50 µM TA and found that TA induced the expression levels of genes that take part in the extrinsic pathway of apoptosis (Figure 6A). Then, we checked the expression levels of TRAIL, DR4, DR5, TRADD, caspase-8, and caspase-3 in the protein level and observed that TA significantly increased the expression levels of these molecules (Figure 6B,C). We confirmed the RNA sequencing results by real-time PCR and found a significant increase in the expression levels of these genes (Figure 6D). Furthermore, we checked the activity of caspase-3 and caspase-7 using the Caspase-Glo 3/7 assay, which also proved the upregulation of caspase-3 activity (Figure 6E).

### 3.7. TA Induces Human TRAIL Expression and TRAIL-Induced Apoptosis in NCCIT Cells

**Figure 7:**
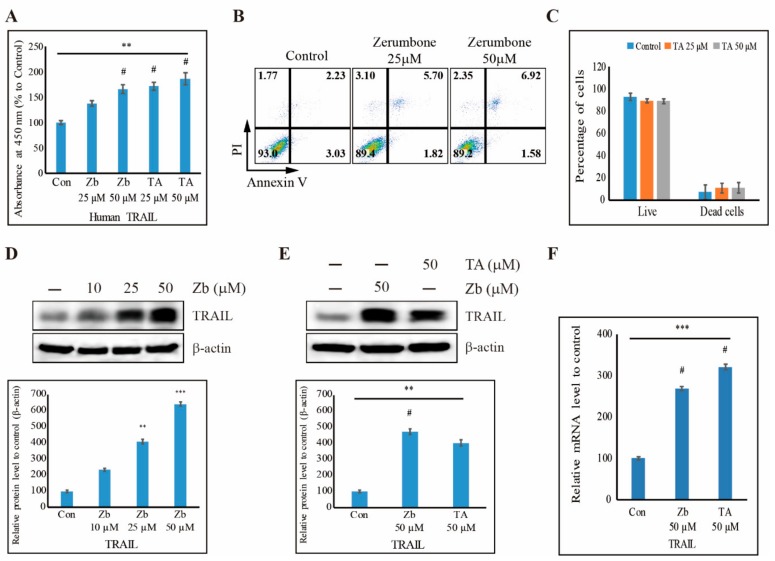
TA induces human tumor necrosis factor-related apoptosis-inducing ligand (TRAIL) expression. (**A**) Human TRAIL assay showing the elevation of human TRAIL with zerumbone (Zb) and TA treatment for 48 h. Control is set to 100. ** *p* < 0.01 (ANOVA test). # The mean difference is significant at the 0.01 level. (**B**) Annexin V-FITC vs. PI staining analysis showing apoptosis induction after treatment with 25 µM and 50 µM Zb for 48 h in NCCIT cells. (C) Graphical analysis of the percentage of apoptotic cells upon control, 25 µM, and 50 µM Zb treatment for 24 h and 48 h. (**D**) Western blotting analysis showing the expression of TRAIL after treatment with Zb for 48 h; the representative expression of TRAIL protein was determined by densitometry and normalized to β-actin. Data are representative of three independent experiments. ** *p* < 0.01 and *** *p* < 0.001 (*t*-test). (**E**) Western blotting analysis showing the expression of TRAIL after treatment with 50 µM Zb and 50 µM TA for 48 h; the representative expression of TRAIL protein was determined by densitometry and normalized to β-actin. Control is set to 100. Data are representative of three independent experiments. ** *p* < 0.01 (ANOVA test). # The mean difference is significant at the 0.01 level. (**F**) Real-time PCR data of mRNA after treatment with TA showing the relative expression levels of TRAIL and normalized to GAPDH mRNA. *** *p* < 0.001 (ANOVA test). # The mean difference is significant at the 0.01 level.

**Figure 8:**
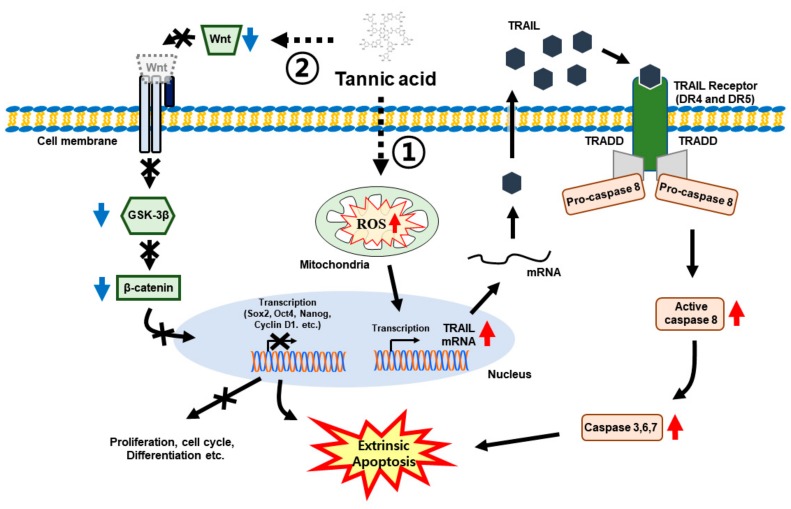
Molecular regulatory mechanism of Wnt/β-catenin signaling, induction of extrinsic apoptosis pathway by natural bioactive TA in NCCIT cells, and role of mROS in TRAIL-mediated extrinsic apoptosis induction with TA treatment.

We showed that TA induced TRAIL-mediated extrinsic apoptosis in NCCIT cells. Following this, we checked whether TA has the ability to induce human TRAIL expression and obtained results suggesting that TA enhanced the human TRAIL expression similar to a commercially available human TRAIL inducer, zerumbone (Zb) (Figure 7A). Zb also induced apoptosis in a concentration-dependent manner (Figure 7B,C) as well as the expression of TRAIL in a significant manner (Figure 7D). Then, we compared the TRAIL activity of Zb with that of the TA treatment in the protein level and found that TA increased the TRAIL expression similar to Zb (Figure 7E). The increased TRAIL activity by TA was confirmed in the RNA level by real-time PCR (Figure 7F). These results suggest that TA inhibited cell proliferation by inhibiting Wnt/β-catenin pathway and promoted TRAIL-induced apoptosis through mROS induction in the NCCIT cells (Figure 8).

## 4. Discussion

The present study demonstrated the induction of mROS and the TRAIL-induced extrinsic pathway of apoptosis by TA in NCCIT cells. The polyphenol TA is well known for its presence in viable diets, which indicates that it is safe for the human body. The concentration of tannin in food varies based on the types of food. A study showed that acetone extracts of cloudberry contain 1600–2400 mg/kg of ellagitannin whereas raspberry and strawberry contain 2500–2600 and 80–180 mg/kg, respectively. Another form of tannin, ellagic acid, was present in pecans (about 310 mg/kg) and walnuts (570 mg/kg) [42]. TA is also known for its inhibitory action against breast cancer stem cells [43]. Many studies were carried out with TA in mouse models where a concentration of 30 mg/kg of TA was used in PSAPP mice [44]. Another study showed that treatment with 10 mg kg^−1^ TA along with diquat in mice induced a non-significant difference in the mice body weight [45]. Targeting these cancer stem cells is a better method of cancer chemotherapy, as it prevents cancer recurrence by attenuating the formation of the cancer stem cells. NCCIT cells are well-known for their ability to differentiate into different cell types and have extensive self-renewal ability [46,47]. Thus, targeting stem cells helps to eliminate the recurrence of cancer. In this study, TA inhibited the proliferation of ES cell carcinoma so that it could not grow further (i.e., its self-renewable activity, as well as its pluripotent behavior). It also inhibited the cancer stem cell markers SOX2, OCT4, and NANOG, further indicating that a natural polyphenol is able to act against cancer cells by mediating cancer stem cells without affecting normal cells [48].

The Wnt/β-catenin pathway is also a well-known molecular cascade in cancer stem cells that contributes to the enhancement of cancer stem cells as well as tumorigenesis [49]. A phytochemical that blocks Wnt/β-catenin in cancer stem cells could be considered the best drug for cancer chemotherapy. Although TA has the ability to block Wnt/β-catenin in cancer cells [50,51], there is no evidence for its inhibition of Wnt/β-catenin signaling in cancer stem cells. Thus, we researched the action of TA in ES cells and found a downregulated expression pattern of the Wnt/β-catenin pathway in both the transcriptional level and translational level. These results indicated that TA is a good drug to treat against cancer stem cells.

We observed the inhibition of cancer stem cell markers and Wnt/β-catenin signaling by TA in NCCIT cells, but the mechanism behind the action was unclear. As such, we investigated the ability of TA to induce cell cycle arrest in ES cells. The growth arrest at any stage of the cell cycle may have led to cell death in cancer stem cells [52]. As expected, we also observed an arrest in the sub-G1 phase in a concentration-dependent manner and an arrest in the S phase in a non-concentration-dependent manner. A molecular analysis of cell cycle markers such as p21, p27, cyclin D1, and CDK4 [53] also provided strong proof of cell cycle arrest, which leads to apoptosis induction in cancer cells. We also observed an early apoptosis in TA-treated cells for 24 h as well as a late apoptosis in the 48-h treatment group. These results suggested that TA is a good therapeutic drug to induce cell cycle arrest and apoptotic cell death in embryonic cancer stem cells.

We found that TA has the ability to induce cell cycle arrest and apoptosis in both cancer cells and cancer stem cells. Therefore, we hypothesized that the mechanism behind these processes was mROS production, as ROS can induce cell cycle arrest as well as apoptosis [54]. Our results also backed our hypothesis, as the TA treatment significantly increased the mROS, suggesting the elevation of mitochondrial activity with TA treatment. An analysis of the mitochondrial membrane potential uncovered downregulated activity with TA, which might have given a hint about the induction of mitochondrial apoptosis with TA treatment, but mitochondrial membrane potential loss does not always take part in the apoptosis process [55]. Then, we investigated the intracellular ATP production by TA, which also saw a decrease in the level of ATP with TA treatment, suggesting that TA did not induce mROS because intracellular ATP is needed for mitochondrial intrinsic apoptosis [56].

From these results, it was evident that TA does not take part in mitochondrial apoptosis, which plays a role in the intrinsic pathway of apoptosis. To confirm this, we checked the BAX/BCL-2 ratio with TA, as it defines the role of the intrinsic pathway of apoptosis [57]; the decrease in the ratio also suggested that TA does not induce the intrinsic apoptotic pathway. To determine the role of the extrinsic apoptosis pathway with TA treatment in NCCIT cells, we performed RNA sequencing for molecular analysis with or without TA. The extrinsic apoptosis pathway is normally activated by a death ligand binding to its DR [58]. The RNA sequencing data showed an increase in the expression of DR TRAIL, which actively takes part in the extrinsic apoptosis pathway by binding to its DRs (i.e., DR4 and DR5) [59]. The extrinsic pathway is initiated by binding the TRAIL to DR4 or DR5; then, it binds to their death adaptor, TRADD, which activates caspase-8. This then activates caspase-3 to promote the extrinsic apoptosis pathway [60]. Our results also showed an enhanced expression of these molecules in the pathway with TA treatment, which clearly suggested the inducement of the extrinsic apoptosis pathway by TA in the NCCIT embryonic cancer stem cells. The elevated expressions of caspase-3 and caspase-7 in the assay also provided strong evidence for our hypothesis. We confirmed the TRAIL induction of TA treatment with another TRAIL inducer, Zb. The human TRAIL assay showed an increase in TRAIL expression with Zb and TA as well as elevated protein and mRNA expressions of TRAIL with TA and Zb. These results suggested that TA induces TRAIL expression to induce the extrinsic apoptotic pathway.

## 5. Conclusions

In summary, the polyphenol TA inhibited the cell proliferation of NCCIT cells, stem cell markers, cancer stem cell pathway, and Wnt/β-catenin signaling. The TA also induced cell cycle arrest and apoptosis in NCCIT cells. The mechanism of TA comprises the induction of mROS, thereby activating the death ligand TRAIL-mediated extrinsic apoptosis pathway in NCCIT cells.

## Supplementary Materials

The following are available online at https://www.mdpi.com/2073-4409/9/2/282/s1, Figure S1: TA inhibits NCCIT cell proliferation and cancer stem cell markers. (A) Crystal violet assay showing the cell proliferation inhibition of NCCIT cells with TA treatment for a time period of 24 h and 48 h. (B) Real-time PCR showing the inhibition of cancer stem cell markers, SOX2, OCT4 and NANOG by TA treatment for 48 h. Data are representative of three independent experiments. Controls are set to 100. *** *p* < 0.001 (*t*-test). Figure S2: RNA-seq data with TA treatment compared with non-treated control, Figure S3: Effects of TA in intrinsic apoptosis pathway, Table S1: RT-PCR primer sequences, annealing temperature, and product sizes, Table S2: q-PCR primer sequences, annealing temperature, and product sizes.
