# Supplementary material for: Tannic Acid Promotes TRAIL-Induced Extrinsic Apoptosis by Regulating Mitochondrial ROS in Human Embryonic Carcinoma Cells
Source: Cells. 2020 Jan 23;9(2):282. doi: 10.3390/cells9020282 (PMC7072125; doi:10.3390/cells9020282)
Supplement: Supplementary file 1 [file cells-09-00282-s001.pdf]

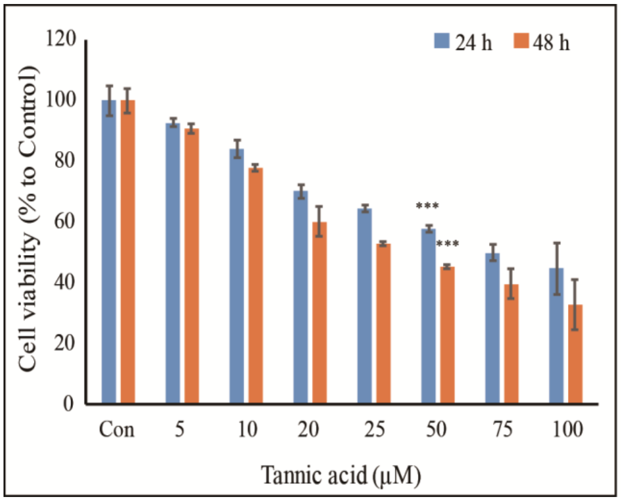

Figure S1. TA inhibits NCCIT cell proliferation. Effects of TA on NCCIT cell viability. Crystal violet assay showing the cell proliferation of NCCIT cells with TA treatment in a concentration-dependent manner with 24-h and 48-h time periods. Data are representative of three independent experiments. ***$p < 0.001$ (t-test).

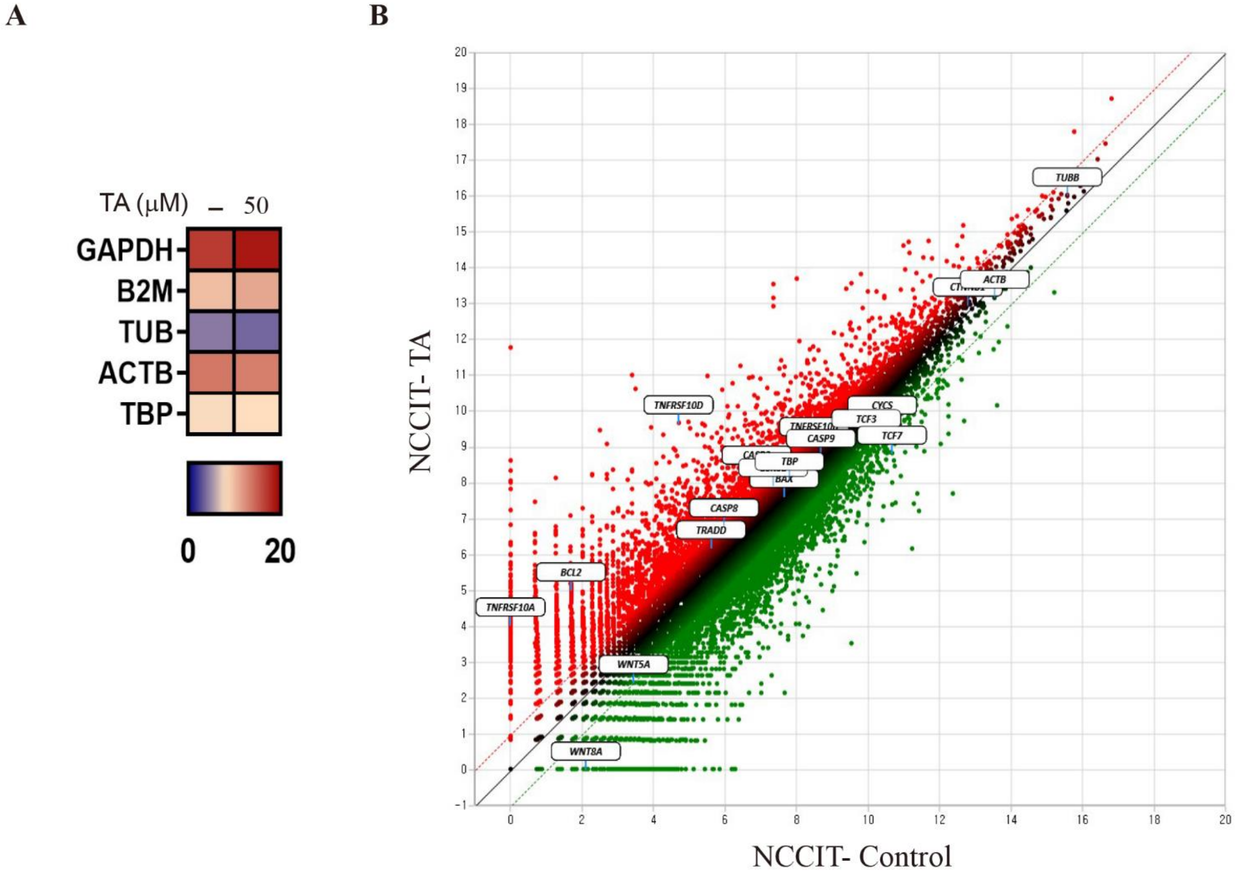

Figure S2: RNA-seq data with TA treatment compared with non-treated control **(A)** Heat map showing the Fold changes relative to the mean expression of housekeeping controls in non-treated and TA-treated NCCIT cells. **(B)** Gene expression analysis by RNA-seq in NCCIT cells are shown by scatter plots.

Each individual colored rectangle is a particular biological function and the color range indicates its predicted activation state: increasing (red) or decreasing (green). Darker colors indicate absolute Z-scores.

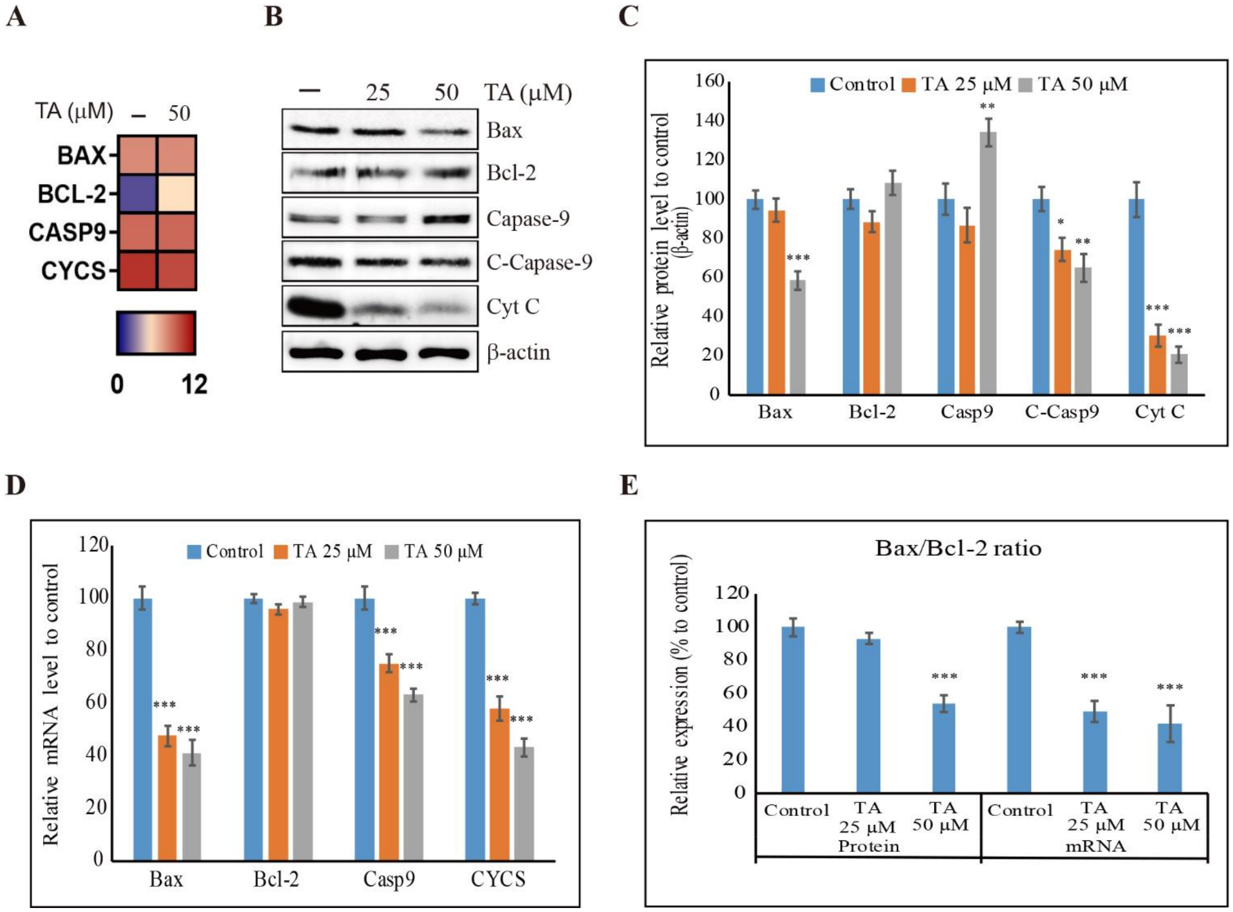

Figure S3: Effects of TA in intrinsic apoptosis pathway. **(A)** Heat map showing the Fold changes relative to the mean expression of intrinsic apoptosis pathway in non-treated and TA-treated NCCIT cells. **(B)** Western blotting analysis showing the expression of BAX, BCL-2, caspase-9, C-caspase-9, and cytochrome C proteins after treatment with 25 μM and 50 μM TA for 48 h. **(C)** Relative expressions of proteins were determined by densitometry and normalized to β-actin. Data are representative of three independent experiments. *p < 0.05, **p < 0.01 and ***p < 0.001 (t-test). **(D)** Real-time PCR data of mRNA after treatment with TA showing the relative expression levels of *BAX*, *BCL-2*, *CASP9*, and *CYCS* and normalized to GAPDH mRNA. Data are representative of three independent experiments. ***p < 0.001 (t-test). **(E)** BAX/BCL-2 ratio of protein and mRNA after treatment with TA for 48 h. ***p < 0.001 (t-test).

Table S1. RT-PCR primer sequences, annealing temperature and product sizes.

Sl No	Gene	Annealing temperature (ºC)	Product size (bp)	Sequence (5` - 3`)
**1**	SOX2	55	165	F-ctgcagtacaactccatgac<br>R-gagtgggaggaagaggtaac
**2**	OCT4	55	190	F-actggttcgctttctctttc<br>R-aaggtattcagccaaacgac
**3**	NANOG	55	202	F-ctcctccatggatctgctta<br>R-ggctgaggtatttctgtctc
**4**	GAPDH	55	191	F-aaggccatcaccatcttcca<br>R-acgatgccaaagtggtcatg

Table 2. q-PCR primer sequences, annealing temperature and product sizes.

Sl No	Gene	Annealing temperature (ºC)	Sequence (5` - 3`)
**1**	WNT5A	58	F- agagtgctcgcatcctca<br>R- ctgcagccagcatgtctt
**2**	WNT8A	58	F- agctctccacccacaaca<br>R- ctccccagatccagccat
**3**	GSK3β	58	F- gtcctgcctgctgaagtg<br>R- aaaccccttttgtggccc
**4**	β Catenin	58	F- gtgggacacagcagcaat<br>R- gacccctgcagctactct
**5**	TCF3	58	F- ggggatgctgtggaaacc<br>R- ccagagaccaaaggcagc
**6**	TCF7	58	F- gcaagaagcaggaagggg<br>R- aggctggtgggtttctca
**7**	TRAIL	58	F- gaagctgaagggtgtcagag<br>R- tgtccttcttccagtgttgc

**8**	DR4	58	F- ggtggtcaattggaggtgtt<br>R- cttttccactcagtccaccc
**9**	DR5	58	F- atgagtcgggaccaaagaga<br>R- ctgtccatagatgggggcta
**10**	TRADD	58	F- ggtgcatcattggggattct<br>R- ctgtaagggctggctgtaag
**11**	CASP8	58	F- atggactgcttcatctgctg<br>R- aagggaagggcacttcaaac
**12**	CASP3	58	F- cgtggtacagaactggactg<br>R- taaccaggtgctgtggagta
**13**	BAX	58	F- atgcgtccaccaagaagc<br>R- cagctgccactcggaaaa
**14**	BCL-2	58	F- tggggtcatgtgtgtgga<br>R- cccagcctccgttatcct
**15**	BCL-XL	58	F- cttctctgctccaccacatc<br>R- aaaactagctgcaagggacc
**16**	CASP9	58	F- ggacatgctggcttcgtt<br>R- tgggtgtttccggtctga
**17**	CYCS	58	F- cccaagcacttctggtgg<br>R- atcacgccattgcactcc
**18**	GAPDH	58	F- cccactcctccacctttgac<br>R- tcctcttgtgctcttgctgg
